# Molecular confirmation and phylogeny of Lassa fever virus in Benin Republic 2014–2016

**DOI:** 10.4102/ajlm.v8i1.803

**Published:** 2019-08-22

**Authors:** Olumuyiwa B. Salu, Ayorinde B. James, Honoré S. Bankolé, Jijoho M. Agbla, Magloire Da Silva, Fernand Gbaguidi, Christian F. Loko, Sunday A. Omilabu

**Affiliations:** 1 Department of Medical Microbiology and Parasitology, College of Medicine, University of Lagos, Lagos, Nigeria; 2 Centre for Human and Zoonotic Virology, Central Research Laboratory, College of Medicine, University of Lagos, Lagos, Nigeria; 3 Department of Biochemistry, College of Medicine, University of Lagos, Lagos, Nigeria; 4 Department of Applied Microbiology and Pharmacology of Natural Sciences, University of Abomey-Calavi, Abomey-Calavi, Benin, Benin; 5 National Laboratory of Public Health, Ministry of Health, Benin, Benin; 6 Department of Pharmacognosy and Pharmaceutical Organic Chemistry, University of Abomey-Calavi, Abomey-Calavi, Benin; 7 De la Pharmacie, du Medicament et des Explorations Diagnostiques, Ministére de la Santé, Cotonou, Benin

**Keywords:** Lassa virus, phylogeny, surveillance, Benin Republic, West Africa

## Abstract

**Background:**

The changing epidemiology of the Lassa virus from endemic areas to other parts of West Africa has been reported. However, there have been no documented Lassa fever transmission chains in the Benin Republic. Two outbreaks of Lassa fever (November 2014 and January 2016) in the Benin Republic were characterised by a high number of deaths (more than 50%) among 27 confirmed and other unconfirmed cases.

**Objectives:**

We report the detection, confirmation and relatedness of the Lassa virus strains from the Benin Republic with other isolates within the West African Sub-region.

**Methods:**

A total of 70 blood samples (16 from 2014 and 54 from 2016) from suspected cases with signs and symptoms suggestive of viral haemorrhagic fever were received for molecular analysis at the Centre for Human and Zoonotic Virology, College of Medicine, University of Lagos and the Lagos University Teaching Hospital. With the detection of the Lassa virus RNA by reverse transcriptase polymerase chain reaction, sequencing and phylogenetic analyses were performed using the Sanger dideoxy sequencing technology platform and the MEGA6 software.

**Results:**

S segments of the Lassa virus RNA genome were detected in 5 (7.1%) of the 70 samples analysed. Sequencing and a phylogenetic tree construction confirmed that the strain of Lassa virus had close relationships with strains previously isolated from Nigeria.

**Conclusion:**

We confirmed the presence of the Lassa virus in the Benin Republic, with 2 strains having molecular epidemiological links with Lineage I and II strains from Nigeria. To reduce the likelihood of outbreaks, there is a need for heightened awareness and strengthened surveillance systems about Lassa fever, particularly in the sub-region.

## Introduction

Lassa fever is an acute and often fatal viral haemorrhagic disease caused by an arenavirus called Lassa virus (LASV), an enveloped bi-segmented negative-sensed single-stranded RNA virus, endemic in parts of West Africa. The illness has caused morbidity of about 300 000 persons and has an estimated death rate of 5000 per annum in West Africa.^1,2,3^ The original LASV strain was isolated in 1969 from a missionary nurse involved in a nosocomial transmission chain from an obstetrical to patient residing in Lassa village, Maiduguri, now Borno state, in Nigeria, who travelled and sought treatment at the Evangel Hospital in Jos, Plateau state, for a septic abortion.^4,5^ In 1974, the reservoir host of LASV was identified: the multimammate rat *Mastomys natalensis*, which is a peri-domestic rodent predominantly found across the sub-Saharan Africa region.^6,7^

The incubation period of Lassa fever ranges between 5 and 21 days with an average of about 10 days.^6^ Transmission is mainly by the ingestion of food or materials contaminated with feces or urine of infected rodents. However, human-to-human transmission of the virus in hospital settings or within the community expands the spread during epidemics.^2,8^ Additionally, global travel, international trading and related commitments are also efficient routes of transmission for highly infectious pathogens, particularly those causing Lassa fever and other viral haemorrhagic fevers (VHFs). By these routes, the movement of infectious agents from endemic countries to new places is a more probable event than has been documented.^9,10,11,12,13^

Recently, LASV was discovered to be maintained by multiple rodent reservoirs other than *Mastomys natalensis.*^1^ The isolation of the virus from the forest dwelling *Hylomyscus pamfi* and ubiquitous *Mastomys erythroleucus* highlights emerging evidence regarding the complexity of the virus’ ecology. The findings add to the growing probability of the emergence of LASV in new environments other than where it currently exists in the West African region.^1^ This is cause for concern regarding the changing epidemiology of LASV within the African continent.

In the Benin Republic, two outbreaks of Lassa fever (November 2014 and January 2016) were characterised by a high number of deaths (>50%) among confirmed cases.^7,14^ We report the detection, confirmation and phylogenetic relationship of LASV from these outbreaks using reverse transcription polymerase chain reaction (RT-PCR) targeting the 5` region of the S segment of the RNA genome and Sanger sequencing of partial fragments of the S segment of the RNA genome.

## Methods

### Ethical considerations

No ethics approval was obtained. This investigation was performed as part of the Lassa fever public health response in the Benin Republic and Nigeria. It was not considered to be research on human subjects, as documented in Otto et al.^15^ All data were completely anonymised before analysis.

### Setting

In 2014, a Lassa fever outbreak was reported for the first time in the Tanguiéta and Cobly communes, Atakora Department, north-west Benin Republic. The chain of infection stemmed from a woman who died from Lassa fever 2 days after the delivery of a baby girl. The baby girl took ill 2 weeks after birth and was cared for at Hôpital de Saint Jean de Dieu, where the outbreak was enhanced by nosocomial transmission and eventual transmission within the community. Within a period of 2 weeks (15 October to 04 November), 16 suspected cases with signs and symptoms of VHF and two laboratory confirmed cases, with a case fatality of 56.3%, were recorded in the country in 2014. This included the deaths of four personnel at the healthcare facility with signs and symptoms of VHF. Due to the high case fatality rate within a short period of time, an alarm for the possible outbreak of the Ebola virus was sounded by the health authorities in the Benin Republic. However, all samples were negative for the Ebola virus; LASV was detected instead.

A resurgence of the epidemic was witnessed in several districts in the central and eastern regions of the Benin Republic in 2016 with reports of 54 suspected cases with signs and symptoms of VHF and 16 laboratory confirmed cases, with a case fatality of about 50%. During this outbreak, the communes of Tchaourou (Borgou department) and Djougou (Donga department) along the Nigerian border were the most affected areas. Since the Benin Republic had never identified a case of VHF, blood samples collected from the people who died and suspected cases were sent to a specialised laboratory, the Centre for Human and Zoonotic Virology, College of Medicine, University of Lagos and the Lagos University Teaching Hospital in Lagos, Nigeria, for VHF investigation.

### Specimen transportation, handling and processing

Blood samples collected from different individuals with signs and symptoms of VHF (16 cases during the 2014 outbreak and 54 cases during the 2016 outbreak) were cold-chain-transported in triple-level packaging to the Centre for Human and Zoonotic Virology and Lagos University Teaching Hospital via the Benin Republic Ministry of Health and the World Health Organization. Universal sample and handling precautions were carried out as recommended by the United States Centers for Disease Control and Prevention.^16^ All specimen transport containers were disinfected with 10% hypochlorite solution in an airtight glove box. Viral agents in specimen aliquots (undiluted and 1:10 dilution) were inactivated in a guanidinium-thiocyanate-based lysis buffer at room temperature for 10 min.

### Nucleic acid extraction and reverse transcriptase-polymerase chain reaction

The viral nucleic acid from inactivated sample aliquots (undiluted and 1:10 dilution) were extracted using a mini spin column RNA extraction kit by Qiagen (Qiagen, Germantown, Maryland, United States) in a Class IIA biological safety cabinet according to the manufacturer’s instructions. After the extraction of viral nucleic acid, S segment of the RNA genome, 3` non-coding region and 5` non-coding region of the nucleic acid of LASV (according to Olschlager et al.^17^), Dengue virus (according to Drosten et al.^18^) and yellow fever virus (using in-house primers) were amplified in discrete RT-PCRs with primers as listed in Table 1. Separate reaction mixtures for Lassa, Dengue and yellow fever viruses were prepared and cycled as described in the One-Step RT-PCR kit by AmbionAgPath-ID protocol (Applied Biosystems, Foster City, California, United States). The reaction was performed using the 9700 Applied Biosystems Thermocycler with the following temperature profile: 50 °C for 30 min and 95 °C for 5 min, followed by 35 cycles of 95 °C for 30 s, 55 °C for 30 s, and 72°C for 30 s with a final extension of 72 °C for 5 min. Subsequently, PCR amplicons were subjected to 1.5% agarose gel electrophoresis with 1X SYBR^®^ Safe DNA gel staining dye (Invitrogen, Carlsbad, California, United States) for 30 min at 120 V/400mA and images of amplicon bands under UV light were taken with a BioDocAnalyze 2.0 (Biometra, Goettingen, Germany).

**TABLE 1:** Primers used for Lassa, Dengue and yellow fever investigation, Lagos, Nigeria, March 2018.

Virus	Primer name	Primer sequence	Amplicon size base pair
Lassa fever virus^24^	36E2	5’GTT CTT TGT GCA GGA MAG GGG CAT KGT CAT 3’	~ 320
	LVS-339-rev	5’ ACC GGG GAT CCTAGG CAT TT 3’	
Dengue fever virus^25^	DenS	5’GGA TAG ACC AGA GAT CCT GCT GT 3’	79
	DenAs	5’ CAT TCC ATT TTC TGG CGT TC 3’	
	DenAs+	5’ CAG CAT CAT TCC AGG CAC AG 3’	
Yellow fever virus (in-house)	YF fwd	5’ ATG GCA CTG TTG TGA TGC AG 3’	405
	YF rvs	5’ AGT TCA AGC CGC CAA ATA GC 3’	

The positive control used for Lassa assays were previously detected Lassa samples from Irrua, Edo State, Nigeria with accession number GU481078 NIG 08-A47 2008 IRRUA, while those for Dengue and YFV assays were both tissue cultured inactivated samples all from the Virology Unit Laboratory of the Bernhard Nocht Institute of Tropical Medicine, Hamburg, Germany through our collaborations.

### Sanger sequencing and phylogenetic analysis

The specific amplicon band size (320 bp) for LASV was purified using the Jena Bioscience gel extraction kit (Jena, Germany). Purified PCR products were sequenced using 3130xl Applied Biosystems Genetic Analyzer at Genewiz Laboratories in South Plainfield, New Jersey, United States.

Sequence data in FASTA format of the S segment of the RNA genome of other submitted or published LASV genome sequences particularly from Nigeria, Liberia and Sierra Leone were downloaded from the National Center for Biotechnology Information. Downloaded sequences were aligned using the MUSCLE tool of MEGA6 software.^19,20^ The Tamura 3-parameter (T92) model was used to deduce the phylogeny of the strains. A phylogenetic tree was constructed using the maximum-likelihood method. Evolutionary rate differences among sites (*+G*, parameter = 0.5024) were determined with a discrete Gamma distribution and the evolutionary invariability allowed for some sites was estimated to be *+I* (36.3524% sites)^21^ using the variation rate model. The consistency of each node on the phylogenetic tree was verified by bootstrapping with 1000 replicates.

## Results

### Reverse transcriptase-polymerase chain reaction amplification and Agarose gel analysis of the Lassa, yellow fever and Dengue viruses

**FIGURE 1:**
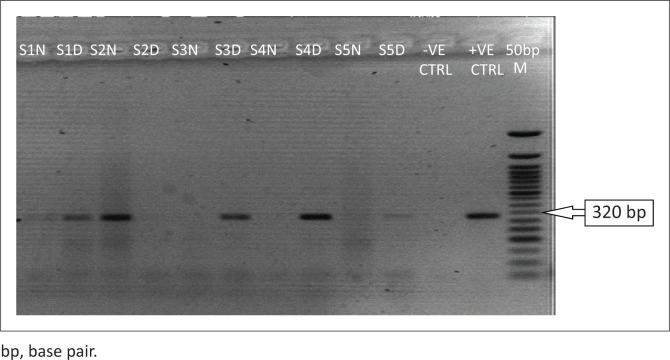
Reverse transcription polymerase chain reaction detection of S gene fragment of the Lassa virus, Lagos, Nigeria, March 2018. The gel lanes represent neat (undiluted, N) and 1:10 dilutions (D) of the RNA extracts used. Three Nigerian outbreak samples representing lanes S1–S3 (accession numbers: MF317933-35) were run alongside Benin Republic outbreak samples (S4–S5). RNase/DNase free water was used as a negative extraction control (-VE CTRL) while a 2008 outbreak positive sample (GU481078_NIG_08-A47_2008_IRRUA) was used as a positive control (+VE CTRL).

**FIGURE 2:**
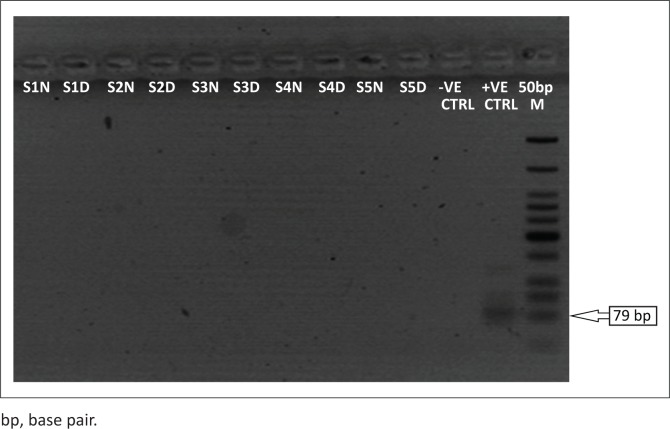
Reverse transcription polymerase chain reaction detection of Dengue virus, Lagos, Nigeria, March 2018. The gel lanes represent neat (undiluted, N) and 1:10 dilutions (D) of the RNA extracts used. Three Nigerian outbreak samples representing lanes S1–S3 (accession numbers: MF317933-35) were run alongside Benin Republic outbreak samples (S4–S5). RNase/DNase free water was used as negative extraction control (-VE CTRL) while a tissue culture inactivated sample of Dengue virus from the Virology Unit Laboratory of the Bernhard Nocht Institute of Tropical Medicine, Germany was used as a positive control (+VE CTRL).

**FIGURE 3:**
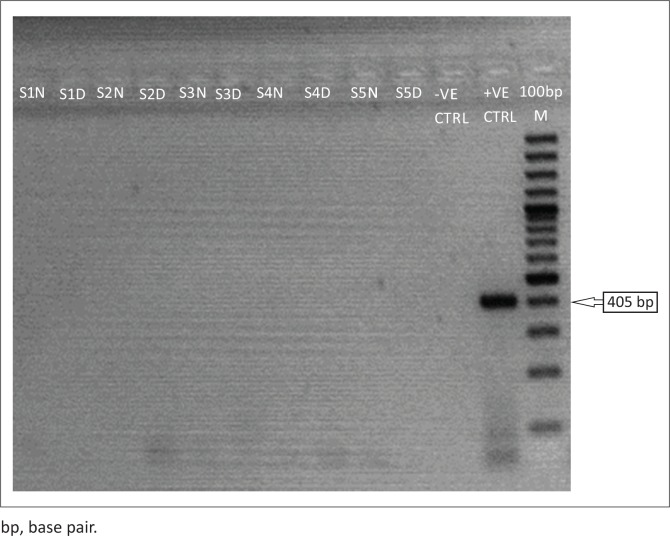
Reverse transcription polymerase chain reaction detection of the yellow fever virus, Lagos, Nigeria, March 2018. The gel lanes represent neat (undiluted, N) and 1:10 dilutions (D) of the RNA extracts used. Three Nigerian outbreak samples representing lanes S1–S3 (accession numbers: MF317933-35) were run alongside Benin Republic outbreak samples (S4–S5). RNase/DNase free water was used as a negative extraction control (-VE CTRL) while a tissue culture inactivated sample of 17D yellow fever strain from the Virology Unit Laboratory of the Bernhard Nocht Institute of Tropical Medicine, Germany, was used as a positive control (+VE CTRL).

Among the 70 samples, 5 (7.1%) were positive for LASV, while none (0%) was positive for both Yellow fever and Dengue viruses. The expected amplicon band size of approximately 320 base pairs (bp) of the S segment of the RNA genome for LASV was detected by the agarose gel electrophoresis analysis^17^ (Figure 1). The detected band size of the LASV amplicons was on par with the positive LASV controls and no band was observed in the negative control lane on the gel picture (PCR-grade water) (Figure 1). However, none of the expected band sizes (~405 bp) and (~75 bp) were detected for yellow fever or Dengue^18^ viruses (Figures 2 and 3).

### Sequencing and phylogenetic analysis of the S segment of the Lassa virus RNA genome

**FIGURE 4:**
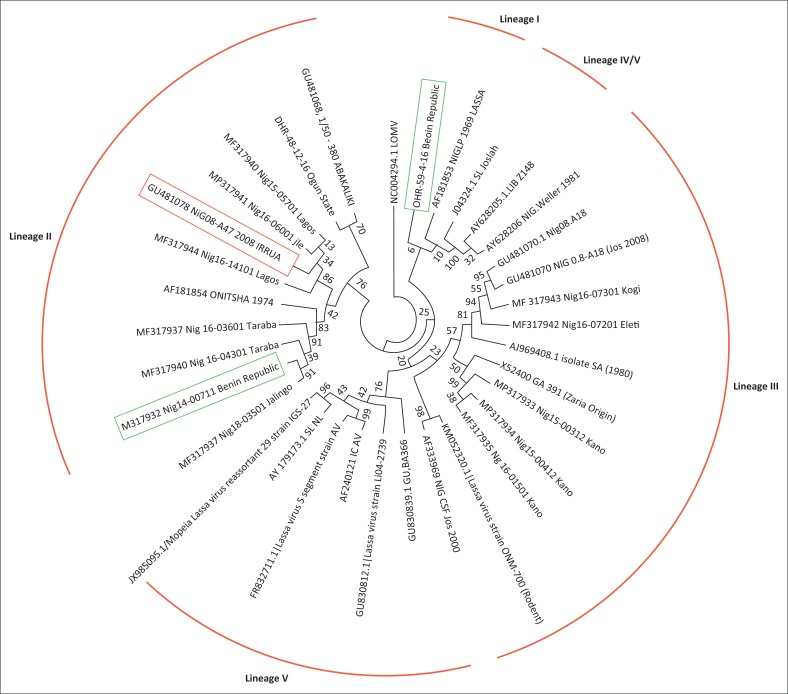
Molecular phylogenetic analysis of the S gene segment of Benin Republic Lassa sequences in comparison with selected Lassa sequences from Nigeria, Liberia and Sierra Leone by the maximum likelihood method, Lagos, Nigeria, March 2018. The numbers at the nodes are bootstrap values. Isolates in green boxes are from the Benin Republic. The isolate in the red box is the positive control of the assay. Lineage I covers the 1969 Lassa LP strain while Lineage II covers strains from Lagos, the eastern states of Nigeria such as the Onitsha strain in 1974, Abakaliki, Irrua, the middle belt and the few northeast central states in Nigeria; Lineage III covers the NIG-CSF-Jos 2000 strain, the western states and northwestern states strains such as GA392 in Zaria, Nigeria; Lineages IV/V cover the Josiah strain of Sierra Leone, and the Liberian strain.

Sequence data of the S segment of the RNA genome of LASV were obtained for 2/5 (40%) of the positive samples. The generated nucleotide sequences of the S segment of the RNA genome of the Lassa strains from the Benin Republic showed relatedness with documented LASV strains particularly from Nigeria. Phylogenetic analysis of the sequences of the 2 LASV strains from the Benin Republic with submitted sequences in the GenBank database showed that each of the strains were closely related to Lineage I that covers the 1969 Lassa LP strain and Lineage II which covers strains from Lagos, the eastern states of Nigeria such as the Onitsha strain in 1974, Abakaliki, Irrua, the middle belt and a few northeast central states in Nigeria as shown in figure 4.

## Discussion

This study confirms the presence of the Lassa virus in the Benin Republic, with 2 isolates having molecular epidemiological links with Lineage-II strains from Nigeria. Large-scale outbreaks of Lassa fever have been reported from Nigeria since 2015 with a frequent and widening geographical spread.^22^ Neighbouring countries are also at risk, because the types of rodents that harbour and spread the virus are found throughout the West African sub-region.^3,6,7^ The Lassa fever virus has been endemic in Nigeria, Sierra Leone and Liberia for decades; however, proven and imported cases have been reported in Ghana, the Central African Republic, Guinea, Cote d’Ivoire, Senegal, Mali and Togo.^22^ The Benin Republic reported the first few cases of confirmed Lassa fever as evident from our laboratory findings during the 2014–2016 outbreaks. It is in no doubt that Lassa fever is becoming a regular global health burden with immense impact on most of West Africa’s communities.^23^ The emergence of the virus in new communities may be attributed to changes in socio-ecological and climatic conditions, global travel and improved surveillance using molecular biology tools for rapid detection of viral nucleic acids.

Due to the proximity of the Benin Republic to Nigeria and reports of several Lassa fever outbreaks in Nigeria since 1969, it was expected that a case of Lassa fever should have been reported in the Benin Republic earlier than now due to fluidic inter-border travel between the two countries. Furthermore, reports have shown that migration of the Lassa virus strains out of Nigeria over a long period of time might have contributed to the increased diversity of LASV, with non-Nigerian strains exhibiting improved codon adaptation to the human host, greater viral loads, and increased case fatality rates.^24,25^

Our phylogenetic analysis of the partial sequences of the glycoprotein region of the Lassa fever virus shows that the 2014 Benin Republic nucleic acid sequence clustered with the Lily Pinneo strain of 1969, whereas the 2016 sequence clustered with the 1974 Onitsha strain. Our findings indicate that the virus may have existed in the *Mastomys* sp. reservoir in the Benin Republic, which did not affect humans until the 2014 outbreak, highlighting the possibility of the emergence of the virus in the Benin Republic which is possibly not a case of inter-border travel. However, cross border transmission from Nigeria to the Benin Republic and vice versa is also still a possibility. Thus, more surveillance studies are required particularly among rodents in the Benin Republic for a better understanding of the molecular epidemiology of the Lassa fever virus in the country.

### Conclusion

Despite the findings from this study, it is still envisioned that the likelihood of movement of VHFs, particularly LASV, from endemic into non-endemic countries within and beyond the West African sub-region remains a possibility. Increased awareness and surveillance are effective tools in curbing the menace of these agents. Thus, laboratory infrastructure, appropriate facilities, technical proficiency and investigation capacity must be improved for a positive impact on our surveillance mechanisms, diagnosis and identification of infections, clinical case management and the development of new approaches to control Lassa fever outbreaks in the sub-region.
